# An operative approach to address severe genu valgum deformity in the Ellis-van Creveld syndrome

**DOI:** 10.1007/s11832-014-0552-9

**Published:** 2014-01-25

**Authors:** Dennis S. Weiner, Jason C. Tank, David Jonah, Melanie A. Morscher, Amy Krahe, Steven Kopits, William C. Schrader

**Affiliations:** 1 Department of Pediatric Orthopaedic Surgery, Regional Skeletal Dysplasia Clinic, Akron Children’s Hospital, Northeast Ohio Medical University, 300 Locust Street, Ste. 160, Akron, OH 44302-1821 USA; 2 Department of Orthopaedic Surgery, Summa Health System, Akron, OH 44310 USA; 3 Medical Illustrator/Researcher Little People’s Research Fund, Baltimore, MD 21228 USA; 4 Department of Sports Medicine, Akron Children’s Hospital, Akron, OH 44308 USA; 5 International Center for Skeletal Dysplasia, Saint Joseph Hospital, Towson, MD 21204 USA; 6 Department of Pediatric Orthopaedic Research, Akron Children’s Hospital, Akron, OH 44308 USA

**Keywords:** Chondroectodermal dysplasia, Ellis-van Creveld syndrome, Genu valgum deformity surgery

## Abstract

**Background:**

The genu valgum deformity seen in the Ellis-van Creveld syndrome is one of the most severe angular deformities seen in any orthopaedic condition. It is likely a combination of a primary genetic-based dysplasia of the lateral portion of the tibial plateau combined with severe soft-tissue contractures that tether the tibia into valgus deformations. Progressive weight-bearing induces changes, accumulating with growth, acting on the initially distorted and valgus-angulated proximal tibia, worsening the deformity with skeletal maturation. The purpose of this study is to present a relatively large case series of a very rare condition that describes a surgical technique to correct the severe valgus deformity in the Ellis-van Creveld syndrome by combining extensive soft-tissue release with bony realignment.

**Methods:**

A retrospective review examined 23 limbs in 13 patients with Ellis-van Creveld syndrome that were surgically corrected by two different surgeons from 1982 to 2011. Seven additional patients were identified, but excluded due to insufficient chart or radiographic data. A successful correction was defined as 10° or less of genu valgum at the time of surgical correction. Although not an outcomes study, maintenance of 20° or less of genu valgum was considered desirable. Average age at surgery was 14.7 years (range 7–25 years). Clinical follow-up is still ongoing, but averages 5.0 years (range 2 months to 18 years). Charts and radiographs were reviewed for complications, radiographic alignment, and surgical technique. The surgical procedure was customized to each patient’s deformity, consisting of the following steps:

1. Complete proximal to distal surgical decompression of the peroneal nerve
2. Radical release and mobilization of the severe quadriceps contracture and iliotibial band contracture
3. Distal lateral hamstring lengthening/tenotomy and lateral collateral ligament release
4. Proximal and distal realignment of the subluxed/dislocated patella, medial and lateral retinacular release, vastus medialis advancement, patellar chondroplasty, medial patellofemoral ligament plication, and distal patellar realignment by Roux-Goldthwait technique or patellar tendon transfer with tibial tubercle relocation
5. Proximal tibial varus osteotomy with partial fibulectomy and anterior compartment release
6. Occasionally, distal femoral osteotomy

**Results:**

In all cases, the combination of radical soft-tissue release, patellar realignment and bony osteotomy resulted in 10° or less of genu valgum at the time of surgical correction. Complications of surgery included three patients (five limbs) with knee stiffness that was successfully manipulated, one peroneal nerve palsy, one wound slough and hematoma requiring a skin graft, and one pseudoarthrosis requiring removal of hardware and repeat fixation. At last follow-up, radiographic correction of no more than 20° of genu valgum was maintained in all but four patients (four limbs). Two patients (three limbs) had or currently require revision surgery due to recurrence of the deformity.

**Conclusion:**

The operative approach presented in this study has resulted in correction of the severe genu valgum deformity in Ellis-van Creveld syndrome to 10° or less of genu valgum at the time of surgery. Although not an outcomes study, a correction of no more than 20° genu valgum has been maintained in many of the cases included in the study. Further clinical follow-up is still warranted.

**Level of evidence:**

IV.

## Introduction

**Fig. 1:**
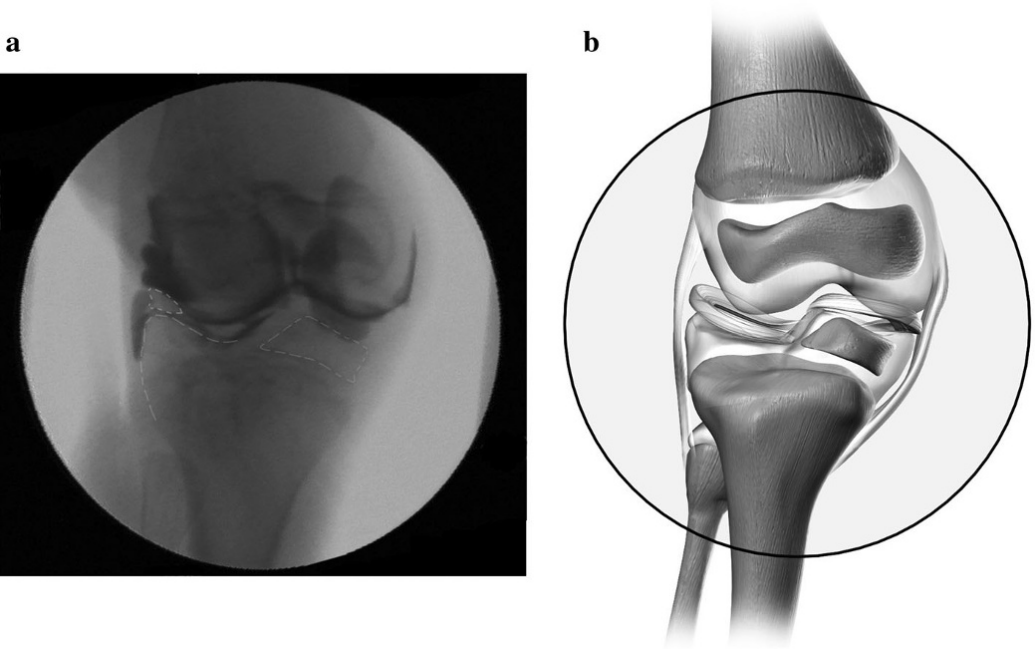
Characteristic “saucer-like” depression of the lateral articular tibial plateau, as seen on **a** anterior-posterior intraoperative arthrogram and **b** representative computer model

Ellis-van Creveld syndrome is a very rare genetic disorder that is seen in most countries, but far more frequently in the Old Order Amish communities of eastern Pennsylvania, likely as a consequence of continuing intermarriage within the communities [1, 2]. The actual incidence in the general population is unknown. The genu valgum deformity seen in Ellis-van Creveld syndrome is one of the most severe angular deformities seen in any orthopaedic condition. It is likely a composite of a primary, genetic-based dysplasia of the lateral portion of the upper tibial epiphysis coupled with progressive and profound weight-bearing changes with growth acting upon the severely valgus-angulated position of the knee [3]. The typical genu valgus deformity consists of lateral subluxation or dislocation of the patella combined with severe contractures of the iliotibial band, vastus lateralis, lateral retinaculum, and joint capsule [4]. Accompanying these are contractures of the lateral hamstrings and of the lateral collateral ligament. There is commonly a lateralized insertion of the infrapatellar tendon. The typical chondro-osseous deformity is a deep “saucer-like” depression of the lateral articular tibial plateau and severe valgus of the proximal shaft of the tibia (Fig. 1) [3–5].

The literature is sparse regarding the surgical treatment of severe valgus deformity in Ellis-van Creveld syndrome, and consists primarily of case reports or small case series, with outcomes rarely reported. In 1954, Metrakos and Fraster reported supracondylar osteotomies in the upper tibia of a 14-year-old patient, but no results were described [6]. In 1979, Zuege et al. [7] reported epiphyseal stapling, but no further details. Pinelli in 1990 reported four cases in the same family, one of which was described to have bilateral supracondylar osteotomies, but no follow-up was mentioned [5]. Pinelli suggested osteotomy for treatment of the valgus deformity and mentioned that frequent recurrences were common and maybe medial epiphysiodesis should be performed [5]. No mention of soft-tissue releases was included. In 1999, Shibata reported six knee surgeries in three patients aged 8, 11, and 12 years in which full soft-tissue release was combined with a closing wedge varus osteotomy and the use of an external fixator [8]. Clearly, Shibata recognized the soft tissue and bony components of the deformity [8]. He reported that the surgical correction obtained was not maintained at final follow-up, but that surgical treatment should begin in childhood or adolescence, in spite of the fact that the surgical procedure did not completely correct the deformity [8]. Eylon et al. [9], in a presentation at the Israeli Orthopaedic Association in 2008, reported on three valgus knees in patients with Ellis-van Creveld syndrome treated by a combined soft-tissue release and bony realignment in a two-staged procedure. They mentioned that in EVC syndrome, corrective high osteotomy does not address the pathology and recurrence is to be expected, and that soft-tissue release combined with bony realignment is necessary [9]. In 2012, Fukuda reported on a single case in an 8-year-old patient who underwent bilateral varus dome osteotomies with K-wire fixation [10]. The deformities recurred and were followed by bilateral varus closing wedge osteotomies of the distal femur with K-wire fixation, combined with soft-tissue release combined with lateral release and medial reefing for the patellar dislocation [10]. They reported improvement at the age of 21 years [10].

In 1982, SK recognized the importance of addressing the soft-tissue deformity and bony deformity through surgery. However, his death preceded the publication of his results. In 2008, DSW acquired the office records of SK, including surgical videos from his research assistant (DJ), and began to follow some of these patients. DSW and his colleagues also began to use the unpublished surgical technique of SK with minor modifications. The purpose of this study is to describe this surgical technique that combines soft-tissue release with bony realignment for correcting the severe valgus deformity that occurs in Ellis van-Creveld syndrome. The significance of the study is that it is a relatively large case series of a very rare condition. It is not an outcomes study, but when available, radiographic measures at last follow-up are reported.

## Methods

This study was conducted under human protocol approval by the Institutional Review Board at Akron Children’s Hospital. Chart, radiographic data, and video analysis from the deceased author (SK) and his research assistant (DJ) were obtained by this hospital system. A retrospective review identified 23 limbs in 13 patients with Ellis-van Creveld that were surgically corrected by radical soft-tissue release and bony osteotomy by two different surgeons (SK, DSW) using a similar approach at two different institutions in the time periods from 1982 to 2001 (11 limbs, six patients) and 2008 to 2011 (12 limbs, seven patients). Seven additional patients were identified from 1982 to 2001; however, they were excluded from this review because sufficient chart or radiographic data was not available. The syndrome is easily identified by clinical and radiographic characteristics, and therefore genetic testing was not utilized. The average age at surgery was 14.7 years (range 7–25 years). Clinical follow-up is still ongoing, but currently averages 5.0 years (range 2 months to 18 years). Since a normal range for genu valgum in Ellis-van Creveld syndrome is not reported in the literature, we arbitrarily defined a successful correction as 10° or less of genu valgum at the time of surgery. Although not an outcomes study, we felt that maintenance of 20° or less of genu valgum at last follow-up was desirable.

All charts were reviewed for operative technique, complications, and subsequent surgeries. Radiographs were used to measure the angle between the femoral shaft and tibial anatomic axis. Radiographic measures, when available, were reported at last follow-up. Nine patients (16 limbs) have had more than 2 years of clinical follow-up.

The surgical procedure performed is described in detail. The extensive surgical exposure enabled direct visualization of the pathoanatomy of the composite soft-tissue contractures and bony deformities.

### Surgical technique

**Fig. 2:**
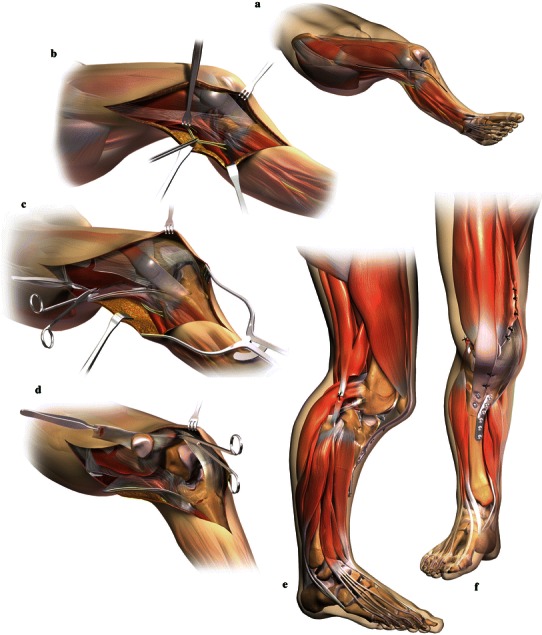
Pictorial representation of the major surgical procedures performed in the operative management of the severe genu valgum deformity in the Ellis-van Creveld syndrome. Below is a brief description of each representation: **a** typical, long curvilinear surgical incision, **b** peroneal nerve mobilization, **c** extensive lateral release, **d** further soft-tissue and patellar mobilization, **e–f** patellar realignment and varus proximal tibial osteotomy

**Fig. 3:**
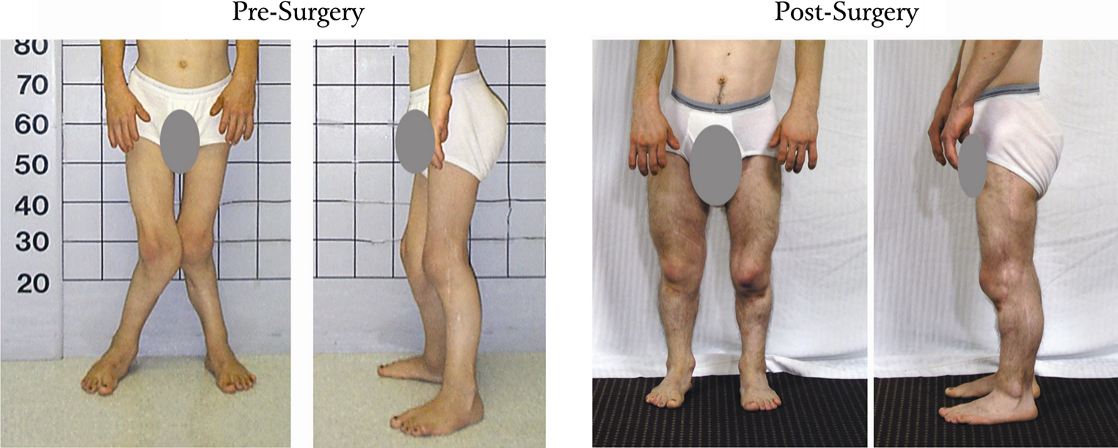
Representative clinical photographs for a single patient taken pre- and post-reconstruction (patient REF #10). The pre-reconstruction photographs were taken when the patient was 16 years of age; post-reconstruction when the patient was 25 years of age or 9 years after surgery. Note that the severe genu valgum, lateral patella, and flexed-knee posture characteristic of the Ellis-van Creveld syndrome were corrected and maintained 9 years post-reconstruction

The surgical procedure performed was customized to each patient’s deformity, with only minor variations. Illustrations of the major surgical procedures performed are presented in Fig. 2. A routine systematic approach was performed in all patients, beginning with a long curvilinear incision (Fig. 2a). This was followed by a complete proximal to distal decompression of the peroneal nerve to avoid stretch injury from the acute correction of the severe valgus deformity (Fig. 2b). The decompression was deemed satisfactory when no points of tension were identified on the nerve after valgus correction. Radical release and mobilization of the quadriceps muscle and iliotibial-band contracture were typically performed after nerve release (Fig. 2c). The contracted lateral soft tissues were addressed by a distal lateral hamstring and lateral collateral ligament lengthening or tenotomies with complete iliotibial-band release. Attention was then focused on proximal realignment of the subluxed or dislocated patella (Fig. 2d). Typically, an extensive lateral retinacular release was performed followed by a vastus medialis advancement and medial patellar femoral ligament plication. If necessary, a patellar chondroplasty was also utilized. Attention was then focused on distal patellar realignment by performing a Roux-Goldthwait transfer or a tibial tubercle transfer, depending upon the patient’s skeletal maturity. The tubercle transfer was anteromedial and internally fixed with compression screws. A proximal tibial varus producing osteotomy with partial fibulectomy was performed to correct the severely abnormal tibial valgus alignment (Figs. 2e–f, 3) [11]. A prophylactic anterior compartment release was performed in all cases. In the early population (SK), triplanar proximal femoral intertrochanteric varus and derotational osteotomies were performed along with proximal iliotibial band releases and gluteus mobilization to permit additional internal rotation of the thigh, which was severely limited in some cases. This has not been deemed necessary in the most recent group of cases. In addition, distal femoral osteotomies were performed in the initial group, but have not been utilized more recently. The average time of the surgical procedure performed by DSW was between 2.5 and 4 h.

All patients were placed in a well-padded, non-weight-bearing, long-leg splint or bivalved cast immediately after surgery. All patients remained in the hospital for at least 48 h and were closely observed, particularly for compartment syndrome and peroneal nerve function. After discharge, patients remained non-weight-bearing until radiographic healing was evident. This generally occurred 3 months postoperatively. Patients were then allowed to be weight-bearing, but were not allowed to participate in running or contact activities. Physiotherapy was usually performed postoperatively in a home setting by parents under instruction or by physiotherapists. Return to full activity generally occurred approximately 6–8 months after surgery.

## Results

In all surgical cases (23 limbs, 13 patients), the combination of radical soft-tissue release, patellar realignment, and bony osteotomy resulted in the correction of 10° or less of genu valgum at the time of surgery. Surgical demographics and radiographic measures for each case included in the study are presented in Table 1. An example of a pre-and postoperative patient is presented in Fig. 3.

**Table 1:** Surgical demographics and radiographic measures for each case included in the study

Patient REF#	Gender	Limb #	Side	MD	Surg year	Surg Age (yrs)	Office Fw-up (yrs)	Pre-Op angle (°)	Last Visit Angle (°)	STR	PF	DF	PT	PN	Other procedures	Complications	Subsequent surgery
1	F	1	L	DSW	2009	16.4	2.4	38	27	Y	N	N	Y	Y			
2	F	2	R	DSW	2009	14.7	3.8	27	19	Y	N	N	Y	Y			Patellar tendon repair’12
		3	L	DSW	2009	14.7	3.7	41	20	Y	N	N	Y	Y			
3	F	4	R	DSW	2009	10.2	3.6	24	5	Y	N	N	Y	Y	HS + patellar tendon lengthening		ROH’10
		5	L	DSW	2009	10.2	3.6	35	50	Y	N	N	Y	Y	HS + patellar tendon lengthening		ROH’10; anticipated revision (by DSW)
4	F	6	R	DSW	2010	25.7	1.9	34	13	Y	N	N	Y	Y	Tubercle osteotomy	Delayed union	
5	F	7	R	DSW	2010	14.7	0.8	17	8	Y	N	N	Y	Y	Tubercle osteotomy		
		8	L	DSW	2011	15.2	0.3	63	20	Y	N	N	Y	Y	Tubercle osteotomy		
6	M	9	R	DSW	2009	15.2	0.7	28	12	Y	N	N	Y	Y		Pseudoarthrosis	ROH + resection’09
		10	L	DSW	2009	15.5	0.4	*	20	Y	N	N	Y	Y			
7	F	11	R	DSW	2009	13.9	0.2	27	10	Y	N	N	Y	Y			
		12	L	DSW	2008	13.0	1.2	26	12	Y	N	N	Y	Y			
8	F	13	R	SK	1982	11.3	18.0	35	7	Y	N	Y	N	Y	Patellar reconstruction	Knee contracture; peroneal n. palsy	ROH + lysis’83, knee manip’83 revision DF’’84; tendon transfer’86
9	M	14	R	SK	1986	15.3	9.9	12	15	Y	Y	Y	Y	Y		Knee contracture	Knee manip + fx’87, ROH’87 DF osteotomy’89, ROH’90
		15	L	SK	1986	15.3	9.9	25	7	Y	Y	Y	Y	Y		Knee contracture	Knee manip + fx’87, ROH’90
10	M	16	R	SK	1998	16.1	9.7	28	0	Y	Y	Y	Y	Y	IT + gluteus mobilized		ROH’00
		17	L	SK	1998	16.1	9.7	29	7	Y	Y	Y	Y	Y	IT + gluteus mobilized		ROH’00
11	M	18	R	SK	2000	17.7	7.9	40	14	Y	Y	Y	Y	Y	IT + gluteus mobilized		
		19	L	SK	2000	17.7	7.9	35	22	Y	Y	Y	Y	Y	IT + gluteus mobilized		
12	M	20	R	SK	1998	7.2	12.1	64	25	Y	Y	Y	Y	Y			fx-ORIF-ROH’05 revision’09 (by DSW)
		20Rv	R	DSW	2009	18.3	1.0	25	14	Y	N	N	Y	Y			
		21	L	SK	1998	7.2	12.1	65	20	Y	Y	Y	Y	Y			Revision’10 (by DSW)
		21Rv	L	DSW	2010	19.1	0.2	20	8	Y	N	N	Y	Y	ROH	Skin breakdown from hematoma	I&D, skin graft’10; ROH’10
13	F	22	R	SK	1992	13.3	2.0	41	10	Y	Y	Y	Y	Y	TFL resection + HS lengthening	Knee contracture	Knee manip’92; ROH’94
		23	L	SK	1992	13.3	2.0	45	7	Y	Y	Y	Y	Y	TFL resection + HS lengthening	Knee contracture	Knee manip’92; ROH’94

*REF* reference, *MD* surgeon, *surg* surgical, *yrs* = years, *Fw-up* follow-up, *STR* soft tissue release, *PF* tri-planar proximal femoral osteotomy, *DF* distal femoral osteotomy, *PT* proximal tibial osteotomy, *PN* peroneal nerve mobilization, *HS* hamstring, *pre-op* pre-operative, *post-op* post-operative, *deg* degrees, *Rv* revision, *F* female, *M* male, *L* left, *R* right, *DSW* Dennis S. Weiner, M.D., *SK* Steven Kopits, M.D., *Y* yes, *N* no, *ROH* removal of hardware, *TFL* tibial femoral ligament, *n*. nerve, *manip* manipulation, *fx* fracture, *I&D* incision and drain, *ORIF* open reduction internal fixation, *IT* iliotibial band

^#^ Number

* Image not available

All surgical complications were documented in Table 1. Early in the series, three patients (five limbs) had knee stiffness. All limbs were treated with knee manipulations (SK), two of which resulted in fatigue fractures of the distal femur. One case of foot drop also occurred early in the series and was salvaged by tendon transfer to restore dorsiflexion of the foot (SK). Peroneal nerve mobilization and decompression were routinely performed in the early series, as well as in the current group. More recent cases have not encountered these complications. One case of proximal tibia nonunion occurred (DSW) and was successfully managed with revision surgery. One patient also required a postoperative blood transfusion (SK). One case of wound breakdown resulting from hematoma formation (DSW) occurred in the revision surgery, as previously mentioned. This was salvaged positively by evacuation and plastic skin closure. One case of acute patellar tendon attrition and insufficiency occurred and was surgically reconstructed (DSW). No clinically significant infections were identified in this series.

**Fig. 4:**
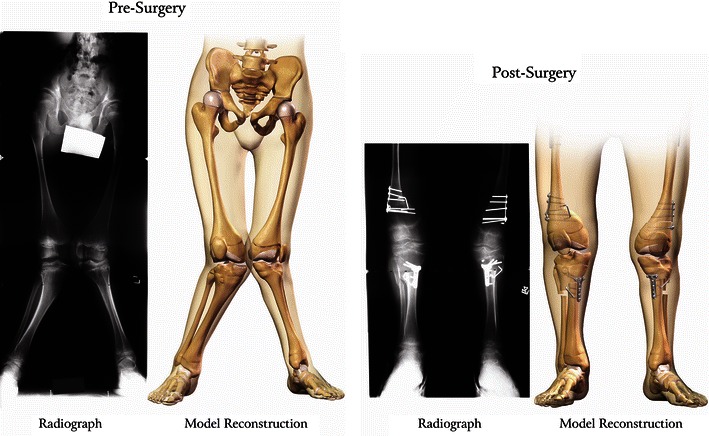
Representative radiographs and computer models of the pathoanatomy pre- and post-reconstruction for a single patient (patient REF # 10). The pre-reconstruction radiographs were taken when the patient was 16 years of age; post-reconstruction 2 months after surgery. The angle between the femoral shaft and tibial mechanical axis was 28° right and 29° left pre-reconstruction, and 0° right and 7° left post-reconstruction

On average, the radiographic angle between the femoral shaft and tibial anatomic axis was 34.3 ± 13.9° pre-reconstruction and 14.9 ± 10.0° post-reconstruction (Table 1). Pre- and post-reconstruction radiographs and computer models of the anatomy are presented in Fig. 4.

Clinical follow-up is still ongoing, but correction of no more than 20° genu valgum was maintained in all but four patients (four limbs). Two of these patients had or are scheduled to have revision surgery. The first patient, a 7.2-year-old male, required bilateral revision realignment surgery at roughly 18 years of age (surgeon DSW). At the time of revision, the genu valgum in each limb was approximately 25 and 20°, respectively. A second patient, a 10.2 year old female at the time of initial surgery (surgeon DSW), will require an additional femoral osteotomy to address persistent deformity. Both these patients presented with severe deformity and repeat surgical correction was not unexpected. To the best of our knowledge, the other two patients (two limbs) with approximately 27 and 22° of genu valgum, respectively, did not seek revision surgery. Both patients had more than 2 years of follow-up and were skeletally mature at their last visit.

## Discussion

This is a relatively large case series (23 limbs, 13 patients) of a very rare condition describing an operative technique that combines extensive soft-tissue release with bony realignment to correct the severe valgus deformity in Ellis van-Creveld syndrome at the time of surgery. The operative technique described was performed originally by one author and later by a second author. All cases (23 limbs, 13 patients) achieved a successful correction at the time of surgery, which was defined as 10° or less of genu valgum at the time of surgical correction. Clinical follow-up is still ongoing, but correction of no more than 20° genu valgum was maintained in all but four patients (four limbs). Two patients had or require revision surgery due to recurrence of the deformity. Further clinical follow-up is still warranted.

Although aggressive soft-tissue releases were performed during this realignment procedure, knee joint instability has not been encountered in this entire population in either the pre- or postoperative period. We believe that the deeply positioned lateral femoral condyle resting within the saucerized lateral tibial plateau imparts knee-joint stability. This was directly observed during surgery.

Decompression of the peroneal nerve is viewed by the authors as essential to avoid permanent nerve dysfunction secondary to acute valgus correction. Peroneal nerve mobilization and decompression were routinely performed in the early series, as well as in the current group. Only one case of foot drop occurred in this population and that case occurred early in the series and was salvaged by tendon transfer to restore dorsiflexion of the foot (SK).

The medial tibia osteochondroma has been noted by various authors [3, 5], and was typical in this series. The exostosis was commonly excised in this series primarily to facilitate fixation of the varus osteotomy of the tibia.

This study is not intended as an outcome evaluation, but is intended to provide a useful surgical approach to a complex soft-tissue and bony deformity. Although the initial results are encouraging, the authors recognize the need to pursue longer follow-up. The authors are also currently evaluating an approach for younger patients (3–8 years old) that combines extensive soft-tissue release with growth modulation techniques.

## Conclusion

The operative approach presented in this study that combines extensive soft-tissue release with bony realignment has resulted in correction of the severe genu valgus deformity in Ellis-van Creveld syndrome of 10° or less of genu valgum at the time of surgery. Although this is not an outcomes study, correction of 20° or less of genu valgum has been maintained in many of the cases included in the study. Further clinical follow-up is still warranted.
